# Very late intubation in COVID-19 patients: a forgotten prognosis factor?

**DOI:** 10.1186/s13054-022-03966-6

**Published:** 2022-04-02

**Authors:** Laurent Camous, Jean-David Pommier, Frederic Martino, Benoît Tressieres, Alexandre Demoule, Marc Valette

**Affiliations:** 1 Intensive Care Unit, Guadeloupe University Hospital, Chemin Chauvel, 97139 Les Abymes,, Guadeloupe France; 2 Institut Pasteur de la Guadeloupe, Morne Jolivière, 97183 Abymes, France; 3 INSERM UMR S_1134, Biologie Intégrée du Globule Rouge, Antilles-Guyane University, 97157 Pointe-à-Pitre, France; 4 Antilles-Guyane Clinical Investigation Center, Inserm CIC 1424, Guadeloupe University Hospital, 97139 Les Abymes, Guadeloupe France; 5 AP-HP, Groupe Hospitalier Universitaire APHP-Sorbonne Université, site Pitié-Salpêtrière, Service de Médecine Intensive - Réanimation (Département R3S), Paris, France; 6 INSERM, UMRS1158 Neurophysiologie Respiratoire Expérimentale et Clinique, Sorbonne Université, 75005 Paris, France; 7 Reanimation Médicale et Chirurgicale, Centre Hospitalier Universitaire de Pointe À Pitre, Chemin Chauvel, 97139 Les Abymes, Guadeloupe France

**Keywords:** Acute respiratory distress syndrome, Mechanical ventilation, COVID 19, Steroid, High flow nasal oxygen therapy

## Abstract

Description of all consecutive critically ill COVID 19 patients hospitalized in ICU in University Hospital of Guadeloupe and outcome according to delay between steroid therapy initiation and mechanical ventilation onset. Very late mechanical ventilation defined as intubation after day 7 of dexamethasone therapy was associated with grim prognosis and a high mortality rate of 87%.

## Dear Editor,

After large therapeutic trials, steroid and tocilizumab are now the cornerstones of COVID 19 pneumonia treatment [1, 2]. Outcome in case of respiratory radiological and clinical worsening under immunosuppressive treatment has to our knowledge not been evaluated. For COVID 19 pneumonia, timing and indications of mechanical ventilation are still heavily debated [3]. In these patients, it has been observed that high flow nasal oxygen (HFNO) could reduce the need for mechanical ventilation and associated morbidity [3, 4]. However, delayed intubation could also be harmful due to Patient-Self Induced Lung Injury (P-SILI) [5]. Here, we aimed to describe the characteristics and outcome of COVID-19 patients who were intubated very late after the onset of a severe form of the disease.

We retrospectively analyzed the characteristics and outcome of the 574 consecutive patients with severe acute respiratory failure and laboratory-confirmed SARS-CoV-2 infection admitted to the ICU of the Guadeloupe University hospital, Pointe-à-Pitre, France, between June 2020 and September 2021. Severe acute respiratory failure was defined as respiratory rate ≥ 25 min^−1^, bilateral pulmonary infiltrates on chest X-ray or CT scan and need for standard oxygen ≥ 10 L min^−1^ to maintain SpO_2_ ≥ 92%. Dexamethasone (DXM) was started for each COVID 19 pneumonia with oxygen requirement. Very late intubation (VLI) was defined as an intubation occurring more than seven days after DXM initiation.

**Fig. 1:**
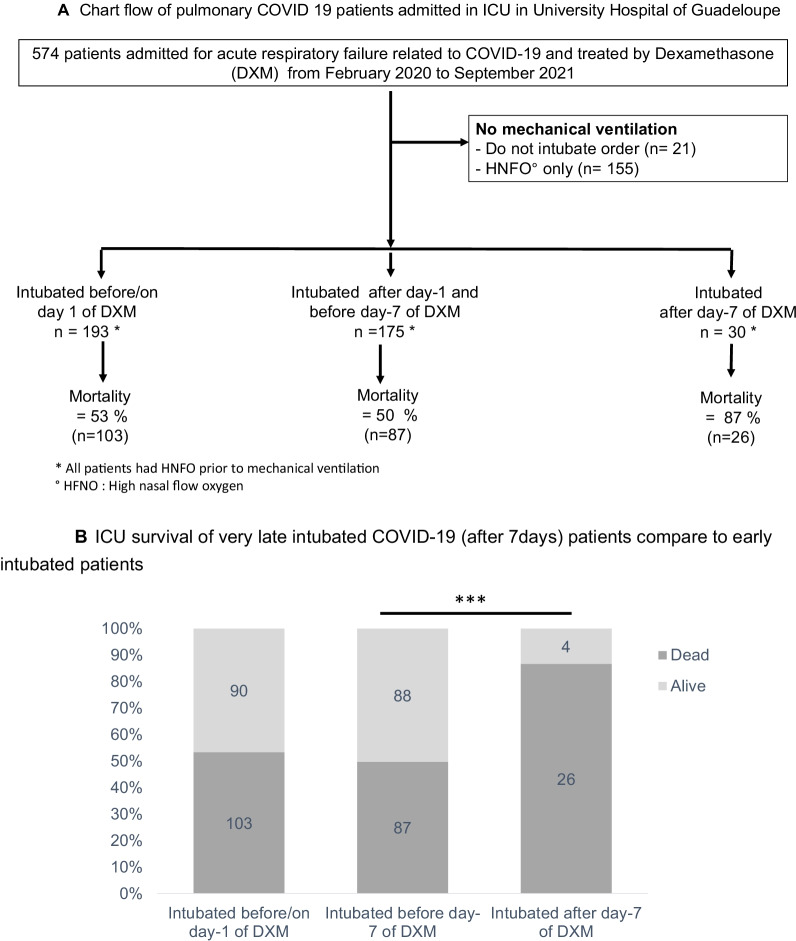
**A** Chart flow of pulmonary COVID 19 patients admitted in ICU in University Hospital of Guadeloupe. **B** ICU survival of very late intubated COVID-19 (after 7 days) patients compare to early intubated patients. Footnotes: All patients were SARS cov-2 PCR confirmed, p-value was calculated using fisher test, *p* < 0.001

Flowchart is shown in Fig. 1A. Patients were classified based on the time between steroid initiation and intubation. Briefly, sub-groups were similar in term of age, sex-ratio and comorbidities (Table 1). Possibly due to a longer time between DXM start and intubation, VLI patients received more adjunctive therapies (awake prone positioning and Tocilizumab) (Table 1). Noninvasive ventilation (NIV) use was similar between groups. Of the 398 patients intubated in the ICU, 30 (8%) met the criteria for VLI. They were mainly male (*n* = 17, 59%) with a median age of 63 years (interquartile range 56–66). VLI patients were intubated 9 days (7–13) after DXM start and 5 days (2–7) after HFNO initiation. All patients had pulmonary samples for microbiological analysis at intubation and only 3 (10%) had nosocomial pneumonia at intubation. Within 24 h following intubation, VLI patients had severe hypoxemia (median PaO_2_/FiO_2_ 86 [70–110] mmHg) with very low respiratory system compliance (median 14 [12–20] ml cmH_2_O^−1^) and severe respiratory acidosis (pH < 7.30 mmHg) (55% of them, *n* = 17). ICU mortality was 87% in VLI patients, 49% in those intubated between two and seven day after DXM initiation and 54% in those intubated on the day of DXM initiation (p < 0.001) (Fig. 1B).

**Table 1:** Characteristics and outcome of COVID 19 patients hospitalized in ICU with delayed intubation 7 days after dexamethasone treatment (≥ 7 days) versus other patients admitted in the same ICU

	All patients (*n* = 556)	HNFO only (*n* = 152)	Intubation ≤ 1 day after DXM initiation (*n* = 196)	Intubation 2 to 7 days after DXM initiation (*n* = 178)	Intubation > 7 days after DXM initiation (*n* = 30)	p
Patient’s characteristics						
Age, years	59 (50–66)	58 (46–66)	60 (50–66)	59 (52–65)	63 (57–66)	0.56
Gender male, *n* (%)	336 (60)	97 (64)	110 (56)	112 (63)	17 (57)	0.55
SOFA	4 (3–7)	3 (2–4)	7 (4–9)	5 (3–7)	5 (3–7)	< 0.01
ROX score on day of HFNO onset, min^−1^	4.2 (3.0–6.0)	6.7 (4.8–8.6)	3.0 (2.2–4.0)	4.2 (3.3–5.2)	6.0 (4.0–7.5)	< 0.01
ROX score at intubation, min^−1^	2.2 (2.0–2.7)	NA	2.2 (2.0–2.8)	2.3 (2.0–2.8)	2.0 (2.0–2.1)	0.45
Comorbidities						
Arterial hypertension*, n* (%)	293 (53)	67 (44)	107 (55)	99 (56)	20 (67)	0.38
Diabetes*, n* (%)	209 (38)	49 (32)	78 (40)	72 (40)	10 (33)	0.18
IMC > 30 kg m^−2^, *n* (%)	291 (52)	71 (47)	116 (59)	89 (50)	15 (50)	0.08
Biology						
White blood cells, G L^−1^	8.6 (6.3–11.3)	8.4 (6.4–10.5)	9.1 (6.8–11.2)	8.7 (1.0–11.5)	8.7 (6.0–11.8)	0.757
D-dimers, ng mL^−1^	1.4 (1.0–2.5)	1.5 (0.9–2.8)	1.2 (0.8–2.6)	1.6 (0.9–2.4)	1.4 (1.0–2.1)	0.93
Adjunctive therapies						
Noninvasive ventilation*, n* (%)	120 (22)	24 (16)	45 (23)	43 (24)	8 (27)	0.66
Awake prone positioning*, n* (%)	154 (27)	71 (50)	18 (9)	53 (30)	12 (40)	< 0.01
Tocilizumab*, n* (%)	66 (12)	33 (22)	5 (3)	19 (11)	9 (30)	< 0.01
Outcome						
ICU mortality*, n* (%)	219 (39)	0 (0)	105 (54)	88 (49)	26 (87)	< 0.01

Results are number *n* (percentage) of for categorical variables and median (q1–q3) for continuous variables. *P* values were obtained using Kruskal–Wallis for continuous variables, and Chi2 or fisher test when appropriate for categorical variables

*HFNO*—high flow nasal oxygen, *DXM*—dexamethasone, *ICU*—intensive care unit, *SOFA*—Sepsis-related Organ Failure Assessment

This is seemingly the first report of COVID-19 patients intubated very late after DXM initiation. Although VLI was uncommon (8% of the intubated patients in our study), it was associated with high mortality. In most studies on timing of intubation in COVID, patients with delayed intubation were actually intubated only 2 days after ICU admission [3, 4] without report on time between DXM start and intubation. The extreme respiratory severity of VLI patients suggests that severe lung damage unrelated to nosocomial bacterial pneumonia occurs prior to intubation. P-SILI [5] and/or a rapid pulmonary fibrotic evolution despite DXM treatment could explain this rapid and severe worsening. Deleterious effect of NIV before intubation on the prognosis of COVID-19 patients has been raised recently [6]. In our study, by definition, VLI patients were exposed to NIV and/or HFNO during a longer period of time, which could have contributed to the generation of P-SILI.

The retrospective and monocentric design without control group of the present study are its main limitations and results should be thus confirmed by other large cohorts.

Based on the grim prognosis of VLI in COVID 19 patients, our study highlights the need for criteria predicting steroid and NIV/HFNO failures in order to avoid delayed intubation.

## Data Availability

The datasets used and/or analysed during the current study are available from the corresponding author on reasonable request.

## Abbreviations

HFNO: High flow nasal oxygen
NIV: Noninvasive ventilation
ICU: Intensive care unit),
DXM: Dexamethasone
VLI: Very late intubation
